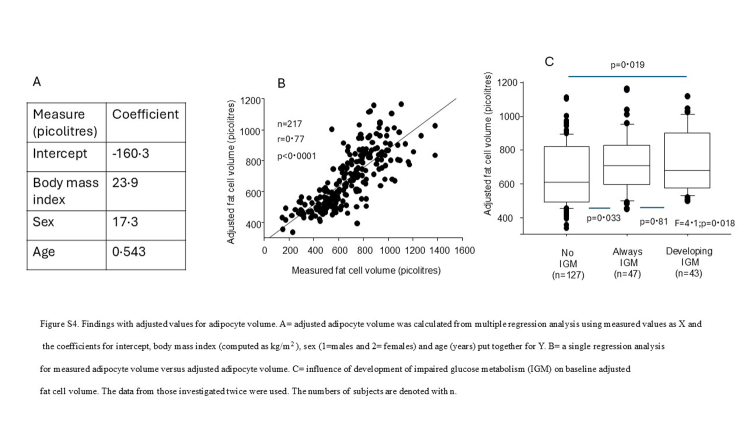# Adipose cellularity and long-term development of impaired glucose metabolism: Swedish cohort study from 1988 through 2016

**DOI:** 10.1016/j.ebiom.2026.106299

**Published:** 2026-06-02

**Authors:** Peter Arner, Thorkild I.A. Sørensen, Daniel P. Andersson

**Affiliations:** a Department of Medicine-H7 at Karolinska Institutet, C2:94 Karolinska University Hospital Huddinge, 14186, Stockholm, Sweden; b Department of Endocrinology, C2:94 Karolinska University Hospital Huddinge, 14186, Stockholm, Sweden; c Novo Nordisk Foundation Center for Basic Metabolic Research, Faculty of Health and Medical Sciences, University of Copenhagen, Blegdamsvej 3B, Copenhagen, 2200N, Denmark; d Department of Public Health Sciences, Faculty of Health and Medical Sciences, University of Copenhagen, Øster Farimagsgade 5, 1353K, Copenhagen, Denmark

**Keywords:** Adipocyte volume, Adipocyte number, Impaired fasting glucose, Type 2 diabetes

## Abstract

**Background:**

Adipocyte volume and number form adipose tissue cellularity, and adipocyte volume relates to impaired glucose metabolism (IGM). We studied how the cellularity relates to future long-term IGM development.

**Methods:**

We investigated 1014 participants living in Stockholm, Sweden 1986–2016 for abdominal subcutaneous adipocyte volume and number and IGM (impaired fasting glucose (≥6.1 mmol/l) and/or type 2 diabetes). A re-examination was conducted 2018–2022 on 241 participants (after on average 15 years); 127 remained without IGM, 47 had developed IGM and 43 remained as IGM. We compared baseline adipocyte volume and number for the three groups by analysis of variance and studied the odds ratio for future IGM as compared with always being healthy by logistic regression, correcting for relevant co-factors.

**Findings:**

Subcutaneous adipocytes were 20% larger in those having IGM from start or developed it later compared with those without IGM throughout (p = 0.005–0.03). Subcutaneous adipocyte number was similar in all three groups (p = 0.26). By comparing upper and lower tertiles for adipocyte volume/number the odds ratio for developing IGM was 2.8 (95% confidence interval CI; 1.2–6.5) for large adipocytes and 0.8 (0.4–2.0) for many adipocytes. Large adipocytes as a risk factor for future IGM was independent of sex, age, observation time, waist-to-hip ratio, body fat, physical activity or undergoing bariatric surgery, but not of insulin resistance measure, which strongly associated with adipocyte volume (r = 0.65).

**Interpretation:**

Adipocyte volume, but not number, associates with risk of long-term development of IGM. Large adipocytes confer this increased risk, maybe through insulin resistance.

**Funding:**

The Stockholm County Council (963296, 994175, 986118), the Centre for Innovative Medicine at Karolinska Institutet (986109), and the Swedish Society of Medicine (1001156). The Novo Nordisk Foundation supports the Novo Nordisk Foundation Center for Basic Metabolic Research (grants NNF18CC0034900 and NNF23SA0084103). None of the funding sources had any involvement in the study.

**Research in context**

**Evidence before this study**

Adipose mass is determined by the volume and number of its adipocytes. In cross-sectional studies this cellularity impacts several metabolic conditions.

**Added value of this study**

Enlarged adipocytes confer a strong risk for development of impaired glucose metabolism (IGM) over time, which is independent of several cofactors, which by themselves may influence development of IGM or associate with adipocyte volume. However, there is no association between adipocyte number and IGM.

**Implications of all the available evidence**

As regards adipose cellularity, adipocyte volume, but not number, associates with development of IGM. Large adipocytes independently of several cofactors confer this risk, wherefore avoidance of enlarging or active shrinking of these cells may reduce the risk.

## Introduction

Adipocyte volume and number (collectively constituting adipose tissue cellularity) are the major determinants of the mass of white adipose tissue.^1^, ^2^, ^3^ Dependent on the relationship between volume and number of the adipocytes, two distinct patterns of white subcutaneous adipose cellularity can be defined.^1^ The tissue can be composed of many, but small adipocytes (hyperplasia), or few, but large adipocytes (hypertrophy). Although there are intermediate stages, hypertrophy and hyperplasia are present over the entire individual spectrum of fat mass, and associate independently of the prevailing amount of adipose tissue with metabolic phenotypes and the metabolic function of the adipocytes.^4^ Of the two cellularity components, adipocyte volume has been extensively investigated including the link to type 2 diabetes (T2DM) as reviewed.^5^, ^6^, ^7^ Importantly, large adipocytes are independent risk factors for later development of T2DM,^8^^,^^9^ but these studies did not explicitly address the role of adipocyte number. To fully understand the importance of the composite white adipose cellularity for impaired glucose metabolism (IGM) it is necessary to simultaneously analyse both volume and number as discussed.^1^^,^^3^^,^^4^^,^^10^^,^^11^ Simply correcting adipocyte volume for the concurrent body mass index (BMI), as often done, is not sufficient, because BMI variation may reflect non-fat mass differences and, moreover, there is large inter-individual variations in adipocyte size and number over the entire spectrum of BMI.^12^ In a prospective study we presently investigated the association between white subcutaneous adipocyte volume or number and future long-term risk of development of IGM over time in participants examined twice with on average 15 years in between. Based on our previous findings on adipose cellularity and changes in body weight over time,^12^ we hypothesised that both volume and number of adipocytes are important for development of IGM. Whereas previous studies have demonstrated the association of adipocyte size with IGM in cross-sectional examinations without considering adipocyte number, we addressed the question of the role of adipocyte size as well as number in future development of IGM, which may pave the way forward to prevent the IGM development.

## Methods

### Participants

**Figure S1:**
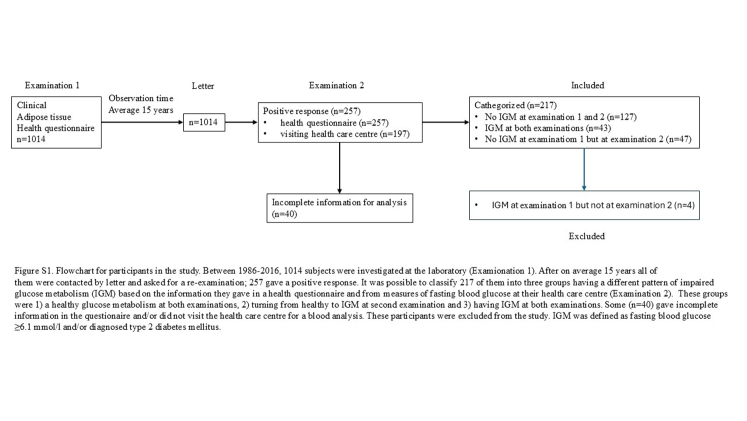


The cohort termed LOSHAT (LOng-term Studies of Human Adipose Tissue) is a convenience sample which has been investigated before as regards the relationship between adipose cellularity and body weight changes over time.^12^ The study design is prospective, based on recruitment over several years and re-examination after different periods of time. Between 1988 and 2016 we recruited 1014 participants living in the Stockholm, Sweden, area for measurements of abdominal subcutaneous adipose cellularity and presence or not of impaired glucose metabolism (IGM). It was defined as either fasting plasma glucose ≥6.1 mmol/l or having a diagnosis of T2DM. All individuals considering themselves to be currently healthy and willing to undertake all investigations were included. None fulfilled the sole exclusion criterium, which was severe medical disorder. About 5% were of a non-European origin. All were body weight stable according to self-report (<±2 kg change in body weight the last 6 months). They came to the same laboratory in the morning after an overnight fast for a clinical/clinical biochemistry examination including homoeostasis model assessment of insulin resistance (HOMA-IR) and in subgroups different measures of percentage total body fat (impedance, Bodystat and/or dual x-ray absorptiometry, DEXA) as described before.^12^^,^^13^ This was followed by an abdominal subcutaneous fat biopsy using prilocaine as anaesthetic agent. They subsequently filled out a health questionnaire in the presence of a nurse as described.^12^ All were contacted by letter for a re-examination between 2018 and 2022 and asked to fill out the same health questionnaire as was used the first time, which included weight/height measures at home. We preferred self-report over using national health registers because the latter do not include measures of fasting glucose. They were instructed to visit their primary health care centre for measures of fasting plasma glucose and body weight. The relationship between self-reported and measured body weight at baseline by linear regression gave r = 0.997, slope 1.0 and intercept not different from zero. In a small subgroup (n = 41) we also obtained visceral (omental) adipose tissue in connection with elective abdominal surgery performed a short while after the examination at our own laboratory and described recently.^12^ Obesity (body mass index, BMI, ≥30 kg/m^2^) was present among 85% of the operated persons of whom 50% were referred because of gall bladder disease and the remaining because of bariatric surgery. At baseline 150 individuals had IGM. In total, 257 participated in the follow-up (about 25%) of the initially examined people. At follow up, 40 participants gave incomplete information in the health questionnaire or did not visit the health care centre. The latter subjects were not used in the classification or analyses. A flow-chart for the study is given in Figure S1.

### Ethical considerations

The initial study was explained in detail to each participant and his/hers written informed consent was obtained. Four were adolescents (16–17 years old), and we also obtained consent from their parents. The follow up study was explained in detail in the invitation letter, and informed written consent was obtained from each participant when they returned the health questionnaire. Many of the participants have been investigated several times before in other studies at our laboratory, as exemplified^4^^,^^12^^,^^13^ and for each of the studies we obtained approval from the regional ethics committee in Stockholm. The two latest approvals allowed us to put together all data from previous examinations at baseline and make the re-examination described above (register numbers 2018-809-31 and 2025-03186-02).

### Adipose tissue examinations

The methods have been described in detail previously.^12^^,^^13^ The mass of subcutaneous abdominal adipose tissue (in kg) was obtained from a formula based on clinical examinations, presented and validated previously.^12^^,^^13^ In brief, dual energy X-ray absorptiometry (DEXA) was carried out in 368 individuals. A formula was derived for the estimated subcutaneous adipose tissue mass. The relationship between DEXA measured and estimated fat mass gave r = 0.9 by linear regression and the difference between the two measures over a large range of body weight was 1%. Isolated adipocytes were prepared by collagenase treatment. The diameters of 100 cells were determined and used for calculation of the mean volume and weight of the cells using established equations.^14^ The adipocyte volumes between individuals has previously been shown to be normally distributed.^15^ The number of adipocytes was calculated by dividing the weight of abdominal subcutaneous adipose tissue with the mean weight of the adipocytes. Adipose cellularity was presently investigated as volume and number of adipocytes as separate quantities. It is possible to categorise the participants according to type of cellularity (many or few adipocytes as well as large or small adipocytes) as described.^12^ However, the number of individuals in some of these combined categories of cellularity was too small for a valid analysis.

### Statistical analyses

**Figure S2:**
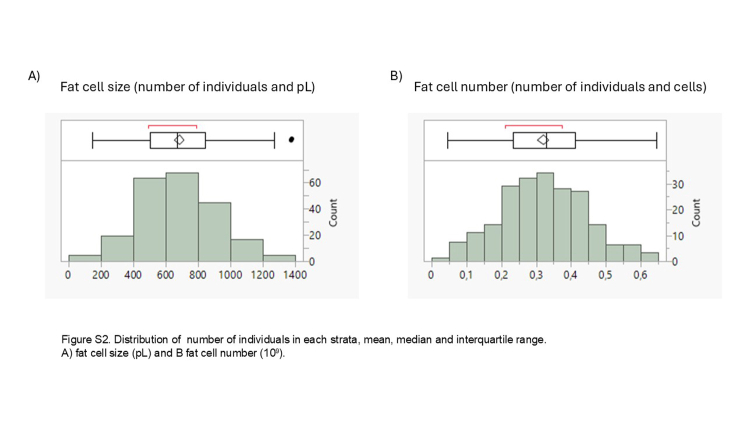


John's Macintosh Project (JMP) student edition 18.2.0 (SAS Institute Inc., Buckinghamshire, UK) was used for data analyses. Values are expressed as mean ± SD (or sometimes range) in the text and tables and as box plots in the figure. Values for adipocyte volume and number were normally distributed (Figure S2). Primary outcome variable was development of IGM over time. Primary independent variables were adipocyte volume and number. The first comparison was between three groups (free of IGM on both examinations, and having IMG already at start, or just at second examination) using chi square tests or analyses of variance (ANOVA) followed by unpaired t-test as post-hoc analysis when ANOVA was significant. As an alternative to ANOVA, two groups were directly compared by t-test using a Bonferroni correction of p-values (i.e. multiplied by three because three groups were examined). The second comparison was between two groups of individuals (always without IGM or developing IGM over time). Odds ratios for developing IGM were analysed. We did not consider it as valid to use imputation of data for the large number of non-attending people. Therefore, odds ratios were calculated for recorded data only. Cox regression analysis was not used because we had no information about the exact time of onset of IGM. In simple models, the odds ratios of IGM when having large or small and many or few adipocytes was assessed. In logistic regression models with IGM as outcome, the odds ratios associated with number of adipocytes or their volumes was determined in the presence of the following variables as cofactors namely, sex, age and observation time, initial BMI or % body fat (by impedance), waist-to-hip ratio, physical activity (sedentary/active), undergoing bariatric surgery and insulin resistance. Each cofactor was implicated as having a possible influence on adipose cellularity and/or development of IGM. Initially, they were compared with adipocyte volume one at a time. We also used a more complex model putting three cofactors together with adipocyte volume. To secure valid output from the regression analyses, the total number of variables put together in one model is determined by the number of cases (turning to IGM) divided by ten, i.e. a maximum of four variables. Finally, in some cases values were compared by linear regression. All statistical tests were two-sided, and p < 0.05 was considered statistically significant. Prior to terminating the inclusion of participants, we made a statistical power analysis for volume and number of abdominal subcutaneous adipocytes using data recorded from 2014 participants in a recent publication.^12^ Those were 682 ± 256 pL for volume and 341 ± 147 million for number. Assuming two equally sized groups of 50 individuals we could detect a 25% difference between groups in volume/number with 80% power at p = 0.05. Because the number of participants always being without IGM was much larger than 50 individuals, we had sufficient statistical power to detect minor difference in cellularity between the two groups with and without IGM.

### Role of funders

Funders did not have any role in study design, data collection, data analyses, interpretation, or writing of the manuscript. None of the authors have been paid to write this article by a pharmaceutical company or other agency.

## Results

About 25% (n = 257) of the participants were willing to be re-examined and all of them returned the questionnaire. One-hundred-ninety-seven participants also visited the health care centre. Based on the information at both examinations, we divided the participants into three groups; those who were without IGM on both occasions (n = 127), individuals who developed IGM at the second examination (n = 47) and those who had IGM on both occasions (n = 43). Thirty individuals changed from no diabetes diagnosis at baseline to a T2DM diagnosis at follow-up. Four had IFG at first examination but not at the second one and were excluded.

The clinical data with participants included in the study and examined twice are recorded in Table 1. Some baseline differences between the three groups were observed. The values for age and observation time were higher in those always having IGM and the frequency of women was higher in those always being without IGM as compared with the other groups. Those developing IGM over time had stable body weight or BMI over time (p = 0.69), whereas the other two groups decreased in these weight parameters over time (p ≤ 0.0001). However, baseline BMI and body fat levels were similar between the three groups. Because body weight development was not the focus of this study, we did not analyse this further. There was no sex difference between the healthy group and the group developing IGM over time (p = 0.42 by chi-square).

**Table 1:** Initial clinical characteristics of participants who completed the first and second examination.

Phenotype	A = no IGM at both examinations	B = develop IGM over time	C = IGM at both examinations	ANOVA or chi-square	Unpaired t-test		
				Overall p value	p value A–B	p value A–C	P value B–C
Sex (male/female)	29/98	14/33	23/20	0.0008	–	–	–
Initial age (years)	43 ± 11	44 ± 9	54 ± 11	<0.0001	0.51	<0.0001	<0.0001
Age at follow-up (years)	59 ± 9	64 ± 9	68 ± 9	<0.0001	0.0009	<0.0001	0.03
Observation time (years)	15 ± 6	19 ± 6	13 ± 7	0.0001	0.0004	0.19	<0.0001
Initial body weight (kg)	88 ± 23	96 ± 19	99 ± 19	0.006	0.03	0.005	0.56
BMI (kg/m^2^)	31 ± 8	33 ± 7	34 ± 8	0.018	0.03	0.02	0.83
Impedance body fat (%)	42 ± 15	44 ± 14	39 ± 17	0.30	–	–	–
BMI change (kg/m^2^)	−2.1 ± 5.6	0.3 ± 6.0	−4.8 ± 6.1	0.0007	0.023	0.015	0.0001
Body weight change (% of initial)	−4.4 ± 15.6	2.0 ± 18.2	−12.8 ± 14.1	0.0004	0.029	0.007	<0.0001
Abdominal subcutaneous fat mass (kg)	2.0 ± 1.1	2.3 ± 1.1	2.1 ± 1.3	0.25	–	–	–
Waist-to-hip (ratio)	0.92 ± 0.09	0.94 ± 0.07	0.97 ± 0.06	0.0016	0.12	0.0004	0.08
Sedentary (yes/no)	33/83	14/23	9/26	0.47	–	–	–
Bariatric surgery (yes/no)	37/90	7/40	7/36	0.07	–	–	–
P-TG (mmol/l)	1.2 ± 1.1	1.9 ± 2.8	1.8 ± 1.4	0.019	0.013	0.05	0.69
P-Total cholesterol (mmol/l)	4.9 ± 0.9	5.4 ± 1.3	5.0 ± 1.3	0.01	0.003	0.42	0.08
P-HDL cholesterol (mmol/l)	1.36 ± 0.39	1.19 ± 0.30	1.24 ± 0.40	0.03	0.01	0.08	0.58

Values are mean ± SD or actual numbers. They were compared by analysis of variance (ANOVA) followed unpaired t-test when ANOVA was statistically significant or by chi square. Participants were characterised into A) having normal glucose metabolism at both examinations, B) having developed impaired glucose metabolism termed IGM (fasting plasma levels ≥6.1 mmol/l) or diagnosed type 2 diabetes mellitus) between first and second examination, or C) having IGM at both examinations. P = fasting plasma. BMI = body mass index. TG = triglycerides. HDL = high density lipoprotein. Changes in BMI or body weight are calculated as values at second minus first examination. The ranges of initial age and follow up time were 21–70 and 5–28 years, respectively.

**Figure S3:**
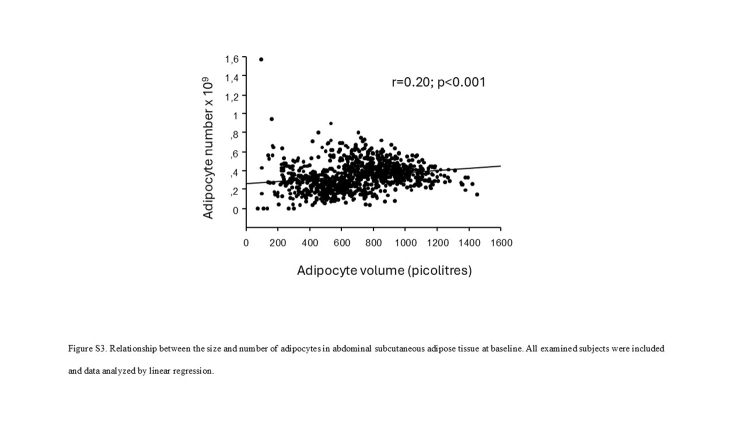


The overall findings with adipocyte volume and number for the whole cohort are depicted in Figure S3. Volume and number were positively correlated but the relationship was weak with adjusted r^2^ of 0.04 suggesting that the two components of adipose cellularity are only weakly, but positively related to each other.

**Fig. 1:**
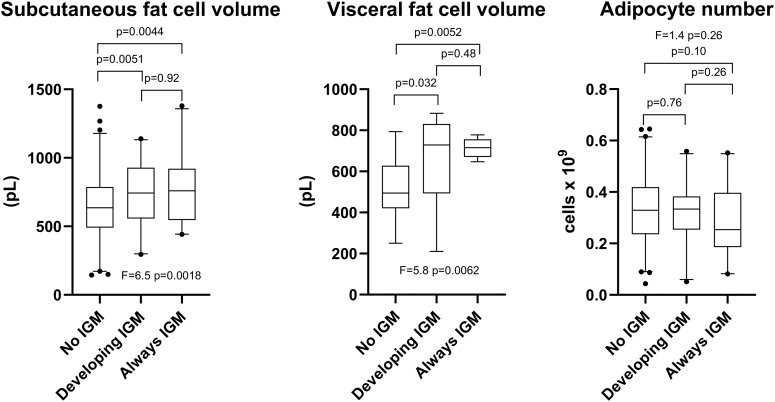
Relationship between glucose development over time and adipose cellularity (volume or number of adipocytes). Data are box plots (whiskers 2.5 to 97.5 percentile) and compared by analysis of variance followed by unpaired t-test when appropriate. Number of individuals for No IGM, Developing IGM and Always IGM are 127, 47, and 43 for subcutaneous adipocyte volume, 28, 7, and 6 for visceral adipocyte volume, and 122, 47, and 43 for adipocyte number, respectively. The participants were divided into three groups as follows, always normal glucose metabolism (no IGM), developing IGM over time (fasting plasma glucose ≥6.1 mmol/l) and/or diagnosed type 2 diabetes mellitus) between the two examinations or having IGM at both examinations. A depicts abdominal subcutaneous adipocyte volume, B is visceral (omental) adipocyte volume and C is abdominal subcutaneous adipocyte number.

We further examined adipose cellularity set in relation to development of IGM over time (Fig. 1). The baseline subcutaneous adipocyte volume among those without IGM was about 20% smaller on average than the corresponding volumes for the other two groups (those having IGM from the start or developed IGM over time). The latter two groups had almost the same mean volumes, about 750 pL In the small group of participants with visceral (omental) adipocyte volume (n = 41), the findings were almost the same as for the subcutaneous region, except that the cells from participants who had no IGM throughout were about 50% smaller than in the two groups having IGM. A quite different picture was observed for abdominal subcutaneous adipocyte number. Number of adipocytes were on average about 300 million and there were no important differences between any of the three groups.

We made additional investigations of the three groups in Fig. 1. Using t-test the difference in subcutaneous adipocyte volume between the healthy ones and each of the other two groups remained statistically significant (a Bonferroni corrected p value for significance is p < 0.017). However, the difference between these groups in omental adipocyte size became statistically insignificant. The unadjusted data in Fig. 1 may be influenced by some of the differences in clinical variables between groups at baseline. Therefore, values for subcutaneous adipocyte volumes were also adjusted for age, sex, BMI and intercept for these adjustments (Figure S4). These results were essentially the same as for non-adjusted volume values in Fig. 1. Because the aim of the study was to investigate development of IGM over time, subsequent analyses were focused on subcutaneous adipose cellularity between participants without IGM throughout and those developing IGM over time.

The odds ratio of developing IGM over time was analysed by subdividing the participants into tertiles based on either adipocyte volume or number and comparing upper with lower tertiles (Table 2). Having large adipocytes conferred about 2.8-fold higher risk of developing IGM over time. No elevated risk was, on the other hand, observed for having many versus few adipocytes (the odds ratio was 0.8).

**Table 2:** Odds ratio for developing impaired fasting glucose or type 2 diabetes (IGM) over time having large or many adipocytes initially.

Phenotype	Odds ratio	95% confidence interval	p-value
Large versus small adipocytes for all participants (n = 115)	2.8	1.2–6.6	0.013
Large versus small adipocytes for those not subjected to bariatric surgery (n = 89)	4.7	1.8–12.8	0.0015
Many versus few adipocytes for all (n = 105)	0.8	0.3–2.0	0.68
Many versus few adipocytes for those not subjected to bariatric surgery (n = 74)	1.3	0.5–3.6	0.63

Adipocyte volume and number were ranked and subdivided into tertiles. Upper and lower tertiles were compared with having a normal state always or developing IGM using logistic regression. For volume the intervals for large/small cells were 144–559/787–1379 pL. For number the corresponding intervals were 44–258/374–645 million cells. The number of subjects is denoted by n.

The separate influence of cofactors on adipocyte volume as risk factor for IGM was investigated in Table 3 with focus on one factor at a time, which might have an influence of their own on development of IGM. Besides adipocyte number those factors were, age and observation time, sex, waist-to-hip ratio, body fat, BMI, physical activity, undergoing bariatric surgery or not, and insulin resistance measured as (10)log HOMA-IR. In these models, adipocyte volume was a strong, significant risk factor for developing IGM, except for when examined together with BMI, showing the same high risk, though with a border-line significant odds ratio (p = 0.07) and HOMA-IR (p = 0.97). Besides adipocyte volume, observation time, undergoing bariatric surgery or not, and insulin resistance were additional significant risk factors (p ≤ 0.002). Adipocyte number played no important role as risk factor (p = 0.38).

**Table 3:** Influence of adipocyte volume on development of impaired glucose metabolism (IGM) considered together with different cofactors examined one at a time using logistic regression.

Model	Odds ratio	95% confidence interval	p-value
Adipocyte volume vs number			
Volume (nanolitres)	8.86	1.96–44.97	0.006
Number (billion x 10)	0.87	0.64–1.18	0.38
Adipocyte volume vs observation time			
Volume (nanolitres)	6.52	1.22–37.98	0.03
Observation time (years)	1.11	1.04–1.19	0.001
Adipocyte volume vs sex			
Volume (nanolitres)	9.86	2.16–50.73	0.004
Sex (male or female)	1.76	0.79–3.87	0.16
Adipocyte volume vs physical activity			
Volume (nanolitres)	7.02	1.39–38.60	0.02
Sedentary or active	0.82	0.37–1.89	0.64
Adipocyte volume vs number vs initial age			
Volume (nanolitres)	8.23	1.90–39.09	0.006
Age (years)	1.01	0.98–1.05	0.43
Adipocyte volume vs body mass index			
Volume (nanolitres)	7.83	0.83–79.3	0.074
Body mass index (kg/m^2^)	1.00	0.93–1.07	0.98
Adipocyte volume vs body fat			
Volume (nanolitres)	15.79	1.63–179.54	0.02
Body fat by impedance (%)	0.98	0.94–1.02	0.28
Adipocyte volume vs body shape			
Volume (nanolitres)	7.03	1.31–40.45	0.025
Waist-to-hip (ratio)	1.55	0.01–228.87	0.86
Adipocyte volume vs intervention against body weight			
Volume (nanolitres)	34.50	6.30–217.07	<0.001
No intervention or bariatric surgery	5.89	2.21–17.8	0.0002
Adipocyte volume vs insulin resistance (HOMA-IR)			
Volume (nanolitres)	0.96	0.13–7.0	0.97
10(log) HOMA-IR (units)	19.3	3.1–121.3	0.002

Odds ratio for development of IGM over time was compared with having no IGM at both examinations. The number of subjects in each model is 151–174. HOMA-IR is homoeostasis model assessment of insulin resistance.

The relationship between adipose cellularity and insulin resistance was further examined by linear regression in the participants followed up (Supplementary Table S1). Adipocyte size was a strong, and number a weak, regressor for HOMA-IR.

Additional subgroup analyses were performed on those being free of IGM over time or had developed this condition at second examination. At baseline the two groups did not differ statistically as regards three different measures of percentage body fat (Table S2). Participants undergoing bariatric surgery or not subjected to this treatment were investigated separately in Table S3. For the latter group results were essentially the same as when there was no subdivision. Size was larger in those developing IGM over time, but there was no influence of adipocyte number on IGM. For the bariatric surgery participants, there was no influence of IGM on size or number. However, we had not sufficient statistical power for a valid analysis in these participants.

Finally, we put together adipocyte volume with three additional significant risk factors from Table 3 (initial age, observation time and sex) into a multiple logistic regression model for developing IGM over time (Table 4). Again, adipocyte volume was a significant risk factor for developing IGM. Also, observation time and initial age but not sex were significant risk factors for future IGM.

**Table 4:** Influence of adipocyte volume on development of impaired glucose metabolism (IGM) considered together with three different cofactors examined together with adipocyte volume in logistic regression.

Model	Odds ratio	95% confidence interval	p-value
Adipocyte volume (nanolitres)	6.9	1.1–42.0	0.036
Observation time (years)	1.2	1.1–1.3	0.0002
Initial age (years)	1.06	1.01–1.11	0.011
Sex (male versus female)	0.92	0.36–1.35	0.87

Development of IGM over time was compared with having no IGM at both examinations. Odds ratio for each factor is given. The number of subjects in the model is 151.

## Discussion

Although the adipose cellularity in humans has been considered since long,^16^ its role for development of IGM over longer periods needs to be further explored. Earlier cellularity studies have primarily been dealing with IGM in cross-sectional examinations.^4^^,^^16^^,^^17^ Only the role of cellularity for future changes in body weight/BMI has been studied prospectively.^12^ Herein, we make a clinically important novel observation on cellularity. Although the subcutaneous adipocyte volume has been shown before as being of importance for the development of T2DM over time,^8^^,^^9^ this issue is presently expanded as regards adipose region and impaired fasting glucose levels prospectively. Furthermore, we presently shed new light on the role of adipocyte number for future development of IGM. To the best of our knowledge this issue has not been reported on before. However, there is one previous study on diabetes development where initial adipocyte number was determined, but no detailed interpretation and discussion of this outcome were provided by the authors.^9^ They showed in one of seven used statistical models that adipocyte number was a risk factor for future diabetes development. As mentioned below this finding is quite different from ours and we do not know why data deviate. Nevertheless, it is essential to consider size and number separately in adipose cellularity studies because if fat mass is not fixed the two factors are only partly dependent of each other as discussed.^1^ Previously, we showed a large interindividual variability in size/number of adipocytes over a wide range of BMI or body weight.^12^ In the present data, we show a weak but significant positive correlation between size and number with a large variation above and below the regression line.

It is presently observed that whether IGM is developed over time or present at baseline, adipocytes are enlarged to the same extent in comparison to what is found when there is no IGM throughout the follow-up period. The finding with always having IGM agrees with a previous study on individuals with T2DM without obesity.^18^ Their subcutaneous fat cells were larger than in healthy individuals. An additional new observation in the present study is that enlarged fat cells are important for future development of IGM, suggesting an early role of adipocyte volume in development of IGM. This is in line with studies on relatives to patients with T2DM showing enlarged subcutaneous adipocytes.^19^, ^20^, ^21^ It might be of value to also examine those having reversal of IGM over time, but, unfortunately, the number of individuals was too small in our study for investigating a general model of the dynamics of IGM over the life course.

Regional differences in adipose cellularity are known to exist, and perhaps most prominent between the subcutaneous and visceral sites as previously demonstrated and discussed.^17^ Although the present data with visceral (i.e. omental) adipocytes were in line with the subcutaneous data, they should be interpreted with caution. The study group was fairly small of different composition from the whole study group, and the outcome was dependent on adjustment variables.

Having adipocytes in the upper tertiles of size was associated with a 2.8-fold increased risk of IGM compared with having adipocytes in the lowest tertile. However, there are many risk factors for developing T2DM as reviewed.^22^^,^^23^ We presently found that when examined together with adipocyte volume; sex, age, observation time, % body fat, physical activity, body shape or undergoing bariatric surgery, adipocyte volume remained as a strong significant risk factor for developing IGM. As for the finding in the analysis together with BMI, the risk of large adipocytes was considerably elevated, but (for unknown reasons) only borderline significant. Furthermore, the role of co-factors such as ethnicity, socioeconomical status or medication was not examined herein. As regards ethnicity, however, no important influence of this factor on adipocyte volume is found among obese women.^24^ In addition, and discussed below, the significance of adipocyte volume for future IGM risk disappeared when examined together with insulin resistance which, in turn, as expected, was a strong risk factor.

Our results with the adipose number component of the cellularity were quite different from what has been found previously for subcutaneous adipocyte number and forecasting of body weight.^12^^,^^25^ Indeed, the number of adipocytes is associated with changes over time in body weight, whether they occur spontaneously or because of bariatric surgery. This does not seem to be the case in the association with current or future presence of IGM. We found similar numbers in participants remaining without IGM as in those having IGM from the beginning or in those developed the conditions over time. Having many or few subcutaneous adipocytes was not associated with future risk for developing IGM in contrast with the observation for large adipocytes. The same was true when adipocyte volume was put together with other possible risk factors for developing IGM over time in various logistic regression models. Thus, our study puts volume in front of number as a more important factor in the pathogenesis of IGM. We have no definite mechanistic explanation for this observation. On the other hand, adipocytes cannot expand indefinitely; they will at some point not store enough lipids to avoid spillover causing ectopic fat disposition. These adipocyte volume related factors may subsequently lead to IGM, as discussed in detail.^5^, ^6^, ^7^^,^^26^, ^27^, ^28^ Clearly the contentions need to be verified by direct investigations.

Future changes in body weight or BMI influence the relative risk for developing T2DM.^29^^,^^30^ Furthermore, the decrease in adipocyte volume after weight loss, induced by bariatric surgery, associates independently with improvement of insulin resistance^31^ which, in turn, is a major risk factor for developing T2DM.^32^ We presently observed different body weight development over time in the three groups that were analysed. However, our logistic regression analysis suggests that adipocyte volume, but not number remains an important risk factor for developing IGM when considered together with baseline BMI, % body fat or body weight. Additionally, in subgroup analyses we found similar baseline total body fat content in participants free of IGM over time or who had developed this condition at second examination.

Bearing in mind the potential role of adipocyte volume for metabolic conditions it seems important to develop easy methods for adipocyte sizing. The present ones are cumbersome and only used in specialised research laboratories. However, we developed recently a fully automatised method using frozen small pieces of abdominal subcutaneous adipose tissue that can easily be obtained in a clinical setting.^33^ Perhaps, this or similar methods can be used on the future for clinical research.

When present and previous data of longitudinal/prospective studies are considered together it is apparent that adipose tissue metabolism is of pathophysiological importance.^34^, ^35^, ^36^ Spontaneous, insulin inhibited, or catecholamine-stimulated lipolysis (breakdown of lipids in adipocytes) associate with future changes in body weight and development of IGM.^37^, ^38^, ^39^ Changes in turnover of adipocytes lipids (the net effect of synthesis and breakdown) associates with future changes in body weight.^40^ Both adipocyte size and number associates with body weight changes.^12^ However, for development of IGM only adipocyte size is important as found earlier for T2DM^8^^,^^9^ and confirmed by the present findings. Several lifestyle factors modify adipocyte size, lipolysis and lipid turnover^1^, ^2^, ^3^ and might be targeted for improvement of adipose tissue metabolic function.

There are some limitations with this study. It was not population-based, but on the other hand, intended population-based studies may not either be representative for the population at large, because they would require people to volunteer for invasive investigations, such as fat biopsy. We made no attempts to subdivide the relatively small IGM cohorts. In theory, the different stages of IGM (i.e. impaired fasting glucose and overt T2DM) may have specific relationships with adipose cellularity. Only about 25% of the participants investigated at baseline also participated in the re-examination. However, we recently demonstrated that in this cohort baseline clinical data are similar between completers and non-completers.^12^ The similarities included obesity frequency, physical activity, and glucose/lipid levels.^12^ The participation rate is similar as in other follow up studies by us and others.^41^, ^42^, ^43^ It is dependent on how data are collected. As an example, Sjöström et al. studied bariatric surgery cases and controls over a 15-year observation period, which is the same mean duration as our time of follow up.^44^ For register data (mortality) compliance was >90% but for data that needed a physical examination (BMI) success rate was only about 10%.^44^ Another example is the adipocyte study of Lönn et al.^9^ in which they were able to reexamine 18% of the initial number of individuals as regards long term healthy state or development of T2DM. The corresponding success rate in our IGM study is very similar, 17%. Although low compliance might bias the true development of IGM there are no obvious reasons why it should influence the assessment of adipose cellularity over a long period of time. We did not use oral glucose testing in the assessment of IGM because it was not performed at health care centres used for the follow up examination. We did not use National Swedish health registers because they do not include measures of fasting glucose. We relied on self-report for defining clinical status at follow up. However, it has been shown before that these measures are reasonably reliable as exemplified.^45^^,^^46^ Additionally, our validation of self-reported body weight showed almost identical results as the measures performed at the health care centres. Bearing in mind all caveats, some exercise should be cautioned in extrapolating our findings to clinical reality. There is a lack of precision in the measures of adipocyte number, IGM is only partly defined, attendance is rather small, and the adjustments for confounding cofactors (for example smoking and exercise) might be incomplete. Therefore, we cannot preclude bias in our conclusions even though we do not find obvious reasons to suspect any bias.

On the other hand, our study has a distinct advantage. Having baseline data on adipose cellularity and relevant cofactors together with the long observation time (about 15 years of average) allowed recognition of a relatively large fraction of cases with newly developed IGM (about 25% of those who initially were without IGM) and how this was prospectively associated with adipose cellularity.

How can large adipocytes cause IGM? Although this study was not mechanistical some speculations can be offered. It is well established that large fat cells associate with an inflammatory state of adipose tissue which, in turn, may case local endocrine and metabolic disturbances so that a state of local insulin resistance is developed as discussed.^5^^,^^6^^,^^47^ Adipose insulin resistance is a causal factor (as other types of insulin resistance) behind T2DM.^48^ We presently found that insulin resistance overrides enlarged fat cells as risk factor for future IGM. In addition, we could confirm the strong relationship between insulin resistance and large adipocytes in the re-examined participants. It is therefore tempting to speculate that the large fat cells cause insulin resistance, which facilitates development of IGM over time. Clearly this speculation must be validated by new direct studies. It should be stressed that our idea is based on prospective studies of abdominal subcutaneous adipose tissue. On the other hand, they are supported by numerous cross-sectional studies on visceral and subcutaneous adipose tissue reviewed before^5^^,^^6^ and exemplified,^49^^,^^50^ The subcutaneous adipose tissue may also have regional impact; the gluteo-femoral fat mass may be protective as reviewed.^51^ Hower, the role of gluteo-femoral adipocyte cellularity for IGM is presently not known.

In summary, regarding the adipose cellularity, adipocyte volume, but not number relates to future development of IGM, which is independent of several co-factors.

## Contributors

PA, TIAS and DPA contributed to the planning of the study and had access to data. PA and DPA analysed the results. PA, TIAS and DPA had full access to all the data in the study and accepted the final version of the manuscript for submission. PA wrote the first version of the paper. PA, TIAS and DPA contributed to further writing and approved the final version of the manuscript. PA, TIAS and DPA were not precluded from accessing data in the study, and they accept responsibility to submit for publication.

## Data sharing statement

All data underlying tables and figures are available upon reasonable request from someone experienced in clinical research. Please contact the corresponding author by email: Daniel.p.andersson@ki.se.

## Declaration of interests

The authors declare that they have no competing interests.

## Figures and Tables

**Figure S4:**