# Supplementary material for: Adipose cellularity and long-term development of impaired glucose metabolism: Swedish cohort study from 1988 through 2016
Source: eBioMedicine. 2026 Jun 2;128:106299. doi: 10.1016/j.ebiom.2026.106299 (PMC13254842; doi:10.1016/j.ebiom.2026.106299)
Supplement: Table S1 [file mmc1.docx]

| Condition | Number of subjects | Adjusted r^2^ | Coeffficient | 95% confidence interval for coefficient | Beta coeffficient | p-value |
| --- | --- | --- | --- | --- | --- | --- |
| Adipocyte volume  (nanolitres) | 213 | 0·43 | 0·89 | 0·75 – 1·03 | 0·65 | <0·0001 |
| Adipocyte number  (times 10^9^ | 208 | 0·06 | 0·67 | 0·31 – 1·02 | 0·25 | 0·003 |

Table S1 relationship between subcutaneous adipocyte volume (nanolitres) or number (times 10^9^) and insulin resistance measured as homeostasis model assessment (HOMA-IR, 10-log units). Baseline values for the participants examined twice are recorded. Linear regression was used.
