# Supplementary material for: Adipose cellularity and long-term development of impaired glucose metabolism: Swedish cohort study from 1988 through 2016
Source: eBioMedicine. 2026 Jun 2;128:106299. doi: 10.1016/j.ebiom.2026.106299 (PMC13254842; doi:10.1016/j.ebiom.2026.106299)
Supplement: Table S2 [file mmc2.docx]

| Groups for  statistical comparison | Impedance | | Bodystat | | Dual X-ray absorptiometry | |
| --- | --- | --- | --- | --- | --- | --- |
|  | Subject  number | % body fat | Subject number | % body fat | Subject number | % body fat |
| Normal over time | 113 | 42 ± 15 | 81 | 38 ± 10 | 50 | 42 ± 11 |
| Developing IGM over time | 28 | 44 ± 14 | 21 | 40 ± 9 | 8 | 44 ± 11 |
| p-value | - | 0.39 | - | 0.39 | - | 0.78 |

Table S2. Baseline body fat content in participants with normal glucose metabolism over time or developing impaired glucose metabolism (IGM) over time from a normal status. The percentage body fat was measured with three different techniques and groups compared by unpaired t-test. Values are mean ± SD.
