# Supplementary material for: Adipose cellularity and long-term development of impaired glucose metabolism: Swedish cohort study from 1988 through 2016
Source: eBioMedicine. 2026 Jun 2;128:106299. doi: 10.1016/j.ebiom.2026.106299 (PMC13254842; doi:10.1016/j.ebiom.2026.106299)
Supplement: Table S3 [file mmc3.docx]

| Measure and type of treatment | Normal glucose metabolism | Developing impaired glucose metabolism | p-value |
| --- | --- | --- | --- |
| Adipocyte volume |  |  |  |
| No bariatric surgery | 550 ± 208 (n=90) | 718 ± 216 (n=40) | <0.0001 |
| Bariatric surgery | 846 ± 172 (n=37) | 928 ± 91 (n=7) | 0.22 |
| Adipocyte number |  |  |  |
| No bariatric surgery | 0.30 ± 0.12 (n=86) | 0.30 ± 0.10 (n=40) | 0.79 |
| Bariatric surgery | 0.41 ± 0.09 (n=36) | 0.45 ± 0.07 (n=7) | 0.29 |

Table S3. Abdominal subcutaneous size (picolitres) or number (times 10^9^) in subgroups. Participants undergoing bariatric surgery on not being subjected to this treatment were investigated. Those having normal glucose metabolism over timed were compared with those developing impaired glucose metabolism over time using unpaired t-test. Values are Mean ± SD.
